# Epidemiology of non-fatal injuries among Egyptian children: a community-based cross-sectional survey

**DOI:** 10.1186/s12889-015-2613-5

**Published:** 2015-12-17

**Authors:** Eman Fawzy Halawa, Abeer Barakat, Hoda Ibrahim Ibrahim Rizk, Eman Mohamed Ibraheim Moawad

**Affiliations:** Department of Paediatrics, Faculty of Medicine, Cairo University, Kasr Al Ainy St., Cairo, Egypt; Department of Public Health, Faculty of Medicine, Cairo University, Kasr Al Ainy St., Cairo, Egypt

**Keywords:** Childhood, Epidemiology, Non-fatal injuries, Egypt

## Abstract

**Background:**

Injuries are a major cause of childhood morbidity and mortality worldwide. We aimed to determine the magnitude and characteristics of child injuries in Egypt and to identify the associated risk factors.

**Methods:**

A community-based, cross-sectional survey was conducted over 27 Egyptian governorates from June to October, 2011. The target population was 1977 households with children aged 0–18 years who had experienced accidental injuries.

**Results:**

In the 6-month period before the investigation, 1576 injuries were reported in 1472 children from a sample population of 1399 households (response rate 70.8 %). Falls (25 %) and burn injuries (20.3 %) were the most common accidental injuries. The incidence of these injuries was significantly higher among boys (57.2 %) than girls and in children aged 2–6 years (70 %) compared with older and younger children. The five main causes of injuries were wounds (30.6 %), fractures (28.7 %), burns (20.3 %), swallowing a foreign body (8.4 %) and accidentally ingesting a poison (7.8 %). Injuries from drowning (n = 27), animal bites (n = 22) and sunstroke (n = 20) mostly occurred in rural children, accounting for 65 %, 54.4 % and 52 %, respectively, of all injuries in rural children. Home and its immediate surroundings (64.4 %) was the most common setting for injuries. Maternal age, education and working status were also associated with childhood injuries (p < 0.05). Children of second and third birth order were at higher risk for injuries (p < 0.0001).

**Conclusions:**

Childhood injuries account for a substantial healthcare burden in Egypt. Our findings emphasise the importance of developing national preventive programs designed to reduce the incidence of childhood injuries.

## Background

Today, injuries remain a major social and health issue, particularly because substantial achievements have been made in the prevention and treatment of infectious diseases [1]. Accidental injuries constitute the leading cause of death in children and young adults. Worldwide, over 875,000 children aged ≤18 years die annually as a result of injuries, mostly in low- and middle-income countries (LMICs), and injuries account for 13 % of the total morbidity among children aged ≤15 years [2]. Children who survive their injuries may require continuing care for disabilities that impact their health, their education and the livelihoods of their families [3]. Unfortunately, several reports from LMICs have shown an increasing trend of childhood injuries, possibly related to the higher prevalence of these injuries as well as limited resources available to address the problem [3–7].

The incidence of childhood injuries is associated with factors such as age, sex, behaviour and environment [8, 9]. Children of lower socioeconomic status generally have a greater risk of both fatal and non-fatal injuries [10]. Like diseases, injuries follow predictable patterns. Therefore, injury prevention in children should be based on an understanding of the causes and patterns (epidemiology) of different mechanisms of injury [11]. There are few studies on injuries in children from developing countries. In Arab countries, public health systems are generally perceived as being non-productive and are given low priority in national financial planning [12–14]. Despite the resources available in some Arab countries, the development and performance of public health systems are lower than expected, with a continued focus on treatment rather than prevention [12, 15].

The present study aimed to describe the epidemiology of accidental childhood injuries among Egyptian children aged 0–18 years. Our findings may provide basic data for future national injury prevention strategies.

## Methods

### Study design and setting

We used a community-based, cross-sectional survey design, with a convenience sample collected from all Egyptian governorates between June and October 2011. Administratively, Egypt is divided into 27 governorates: the four urban governorates (Cairo, Alexandria, Port Said and Suez) have no rural population. The remaining 23 governorates are subdivided into urban and rural areas. Nine of these governorates are located in the Nile Delta (Lower Egypt), nine are located in the Nile Valley (Upper Egypt), and the remaining five are frontier governorates located on Egypt’s eastern and western boundaries. In mid‐2011, the national population was estimated at 81 million, meaning Egypt has one of the highest population densities in the world. According to the 2006 census, 42.9 % of the total population were urban residents, 51.1 % were male and 10.6 % were under 5 years of age [16].

### Study population

The target population was 1977 households with all children aged 0–18 years who had experienced accidental injuries during the study period (the 6-month period before investigation) per household.

Households were initially selected to represent childhood population in Egypt. It was recommended that a minimum of 1921 children should be surveyed. This sample size was obtained to achieve a confidence level, 95 %, 5 % margin of error and 50 % prevalence of unintentional child injuries (there is no national studies worked on the same age categories and covered the 4 regions of Egypt) with expected response rate 80 %. Sampling employed door-to-door protocol for all households. Children who suffered intentional injuries committed by others (i.e. stab wounds, gunshot wounds, other physical violence or sexual abuse), intentional self-inflicted injuries, injuries in children without a legal guardian, and fatal injuries were excluded from the study.

### Data collection tool and methods

Ninety-four medical undergraduate students were recruited and trained as investigators and interviewers to collect the relevant information from households. Data were collected using a pre-tested, coded, structured interview questionnaire at a household level. Participants were predominantly the children’s mothers (70.8 %). In the absence of the mother, the main carer or other responsible adult member of the household completed the interview. Participants were asked about any history of injuries to their children during the last 6 months. Injuries included bodily lesions resulting from acute exposure to energy in amounts that exceed the threshold of physiological tolerance, or an impairment of function resulting from a lack of one or more vital elements such as water or air [17]. For inclusion in the study, an injury was diagnosed based on one of the following circumstances: (a) if the child was injured and treated with simple medical therapies by parents or another authorised adult; (b) if the injury was diagnosed by a doctor or nurse in a clinical setting; or (c) the child was absent from school or rested for one or more days because of injury. These definitions allowed the reported injuries to reflect cultural variations in the use of such care.

To ensure the quality of the collected data, completed questionnaires were submitted and checked for missing information on the same day of the field survey. Feedback was provided to investigators before the next day’s survey.

After designing the questionnaire, we conducted a small-scale pilot study in four villages in Cairo’s sub-districts. This aimed to train the research staff in managing data collection and entry. After this pre-test, the questionnaire was modified (the content of some questions was changed).

The questionnaire consisted of three parts: Part A covered family particulars such as parents’ age, residence, education, occupation and total number of children. Part B. collected detailed information about the children such as age, sex, birth order, injuries during the past 6 months, location and types of injuries and use of health services. Part C contained questions to assess the knowledge, attitude and practice (KAP) of the children’s primary carers towards first aid measures (defined as the assessments and interventions that can be performed immediately with minimal or no medical equipment) [18] for different childhood injuries. This section comprised 13 simple-choice questions on knowledge of treatment of common childhood emergencies. The questions were developed using a PedFACTs textbook and an instructor’s resource manual published by the American Academy of Pediatrics [19]. Each correct answer was given one point, with no points given for unanswered questions or answers of “Not sure”. Other questions covered the use of traditional procedure-based therapies (defined as therapies that use various techniques, primarily without the use of medication, to provide health care) [20]. The parent/responsible adult completed the questionnaire using parent–child scenarios depicting child injury situations. Children were grouped by age: 0–2 years, 3–5 years, 6–11 years and 12–18 years. In the latter group, the sample size was not large enough (4 %) to provide separate estimates for this age group; so they were combined with the age group below into a larger age-group (6–18 years).

We excluded respondents who had children with unintentional injuries more than 6-month period prior to being interviewed (n = 322). Respondents more than 80 years old (n = 58) were excluded from the analysis because of concerns regarding ability to reliably respond to and interpret questions. All households who didn't live in the same dwelling space and acknowledge a common household head (n = 159) were also excluded. These procedures reduced our sample size to 1438 households. Moreover, thirty-nine respondents were excluded because of missing data.

Ethical approval was obtained from the Institutional Review Board (IRB) of the New Children’s Hospital (Abou el Reesh), Paediatric Specialized Hospital of Cairo University before the study started. Informed consent was obtained from all the participants before they were enrolled in the study.

World Health Organisation (WHO) guidelines for conducting community surveys on injuries were used in planning this study [21].

### Data analysis

Data were prepared in Microsoft Excel 2010 and analysed with SPSS Version 16 (SPSS for Windows, Version 16.0. Chicago, SPSS Inc.). Questionnaire data were entered by the trained investigators. To ensure the validity of the data, 5 % of the questionnaires were re-entered by different investigators, and the results were matched to the previous entries. The questionnaire was originally developed in Arabic and then translated into English. Data were analysed to determine the socioeconomic characteristics of the injured children, types of common injuries, risk factors associated with the injuries, the incidence and distribution of the injuries by sex, age and injury types, as well as the KAP of the carer. Simple frequency tables were used for the qualitative variables, and we calculated simple statistical parameters, such as mean and standard deviation (SD), for the quantitative variables. We have presented key data as figures to highlight the priority issues. Questionnaires with missing values were excluded from the present analysis (2 %, n = 39). The differences between proportions were assessed using the p-value for heterogeneity. Chi square and Fisher’s exact tests were used to estimate differences in qualitative variables. A p-value less than 0.05 was considered statistically significant.

## Results

### General description of the population

**Fig. 1:**
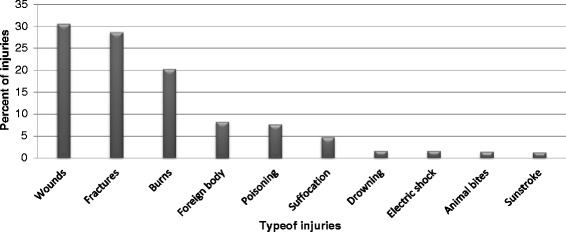
Percent distribution of type of unintentional childhood injuries in the last 6 months

In total, 1576 injuries (Fig. 1) were reported for 1472 children aged 0–18 years during the 6-month study period. The analysis covered 1399 of 1977 possible households (response rate 70.8 %). The responses included 1404 cases (95.4 %) of one injury, 38 cases (2.6 %) of two injuries, 24 cases (1.6 %) of three injuries and 6 cases (0.4 %) of four injuries. General characteristics are presented in Table 1. Over half of the injured children were boys (57.2 %, n = 842), with girls accounting for the remaining 42.8 % (n = 630) of injuries, giving a male to female ratio of 1.3:1. The children’s mean age was 4.4 years (SD = 2.5), with the majority of children being aged 2–6 years (70 %). Most injuries occurred at home (64.4 %), with 8.1 % reported as occurring in a school setting. The major causes of injuries were falls (25 %), burns (20.3 %) and road-traffic injuries (RTIs) (17.2 %) (Table 1). Although RTIs involving pedestrians were the third most common cause of injuries, those involving passengers or drivers in vehicles or on bicycles were uncommon and comprised less than 1 % of all injuries. All injuries reported during the study period recovered with no disability or fatality.

**Table 1:** General characteristics of the study population

Injury characteristics (n = 1576)	% (n)
Fall	25 (368)
Burn	20.3 (299)
Traffic-road	17.2 (253)
Sharp objects	10 (147)
Foreign body swallowing	8.4 (124)
Poisoning	7.8 (115)
Suffocation	4.8 (70)
Drowning	1.8 (27)
Electric shock	1.8 (26)
Animal bites	1.5 (22)
Sunstroke	1.4 (20)
Home	64.4 (948)
Road	26.4 (388)
School	8.8 (130)
Others	7.5 (110)

### Age-based injury patterns

Injury types varied within the age groups (Table 2). Children younger than 2 years of age (n = 125) had higher rates of burn injuries (30.4 %) than other age groups. However, there were no reports of electric shock or sunstroke injuries in this age group. Children in the group aged 2–6 years (n = 1032) comprised the largest group in the study (70 %). Wounds [71.6 % (n = 323)], fractures [67.5 % (n = 285)] and burns [71 % (n = 212)] were the most common injury types in this age group. Drowning (1.7 %) and animal bites (1.4 %) had the lowest occurrence in this age group. The group aged 6–18 years comprised school-aged children and adolescents (n = 315). This group had higher rates of fracture injuries (37.5 %), wounds (31.4 %) and burns (15.6 %). The least reported injuries in this age group were electric shock and sunstroke, with only 2 reported cases of each. In terms of the setting where the injury took place, children younger than 2 years were most likely to be injured at home (n = 120), whereas those aged 6–18 years were more likely to be injured on the road (n = 200) or at school (n = 86) than at home (n = 17).

**Table 2:** Age and sex distribution of non-intentional injuries among study population

Causes of injuries	Total	Age group in years (y)				Sex		
	n = 1576	≤ 2 y n = 125 % (n)	2- <6 y n = 1032 % (n)	6-18 y n = 315 % (n)	P value	Male n = 842 % (n)	Female n = 630 % (n)	P value
Wounds	451	6.4 (29)	71.6 (323)	22 (99)	0.169	19 (281)	11.5 (170)	0.009
Fractures	422	4.4 (19)	67.5 (285)	28 (118)	< 0.001	17 (251)	11.6 (171)	0.269
Burn	299	12.7 (38)	71 (212)	16.3 (49)	0.002	10.9 (160)	9.4 (139)	0.150
Foreign body	124	10.5 (13)	83 (103)	6.5 (8)	< 0.001	3.6 (53)	4.8 (71)	˂0.001
Poisoning	115	11.5 (13)	75.7 (87)	13 (15)	0.056	3.8 (57)	3.9 (58)	0.095
Suffocation	70	8.6 (6)	85.7 (60)	5.7 (4)	0.004	3 (45)	1.7 (25)	0.265
Drowning	27	11.1 (3)	40.7 (11)	48.2 (13)	0.002	1 (16)	0.7 (11)	1
Electric shock	26	0 (0)	92.3 (24)	7.6 (2)	0.040	1 (15)	0.7 (11)	1
Animal bite	22	4.6 (1)	72.7 (16)	22.7 (5)	0.799	0.7 (11)	0.7 (11)	0.521
Sunstroke	20	0 (0)	90 (18)	10 (2)	0.129	0.7 (11)	0.6 (9)	0.825

### Sex and injury characteristics

Girls were statistically more likely to swallow foreign bodies (4.8 % vs. 3.6 %, p = 0.0009) and slightly more likely to ingest a poisonous substance (3.9 % vs. 3.8 %, p = 0.09) than boys. Girls were more likely to sustain these injuries at home (59.2 % vs. 55.3 %) than boys. Boys were statistically more likely to experience falls (10 % vs. 6.3 %, p = 0.02) and wounds (19 % vs. 11.5 %, p = 0.009) than girls (Table 2).

### Time-lapse from injury to medical care

Only 38.4 % (n = 565) of the study population sought medical advice, usually from a primary health care provider (93.5 %). The majority of these cases (61.7 %, n = 349) presented after the first 24 hours of injury, and 20.8 % (n = 118) of the patients presented earlier (usually within 3–6 hours if injury).

### Risk factors

In terms of geographic area, the risk of injuries was higher in the lower Egypt governorates (720 participants) than the urban governorates (520 participants) (51.5 % vs. 37.2 %, p < 0.0001). Upper Egypt and frontier governorates had the lowest distribution of injuries, with only 11.4 % of participants. Children living in urban regions, including the capital central region, had an incidence of injuries 1.4-times higher than those living in rural areas, although, this difference had no statistical significance (with the exception of sunstroke injuries, p < 0.05). Rural children (n = 13) were at greater risk of sunstroke injuries (0.9 % vs. 0.5 %, p = 0.04) than urban children (n = 7).

Maternal age (classified according to the Egypt Demographic and Health Survey, 2008) [22], maternal education, occupation and employment were significant predictors of fracture injuries (p < 0.05). The majority of children were mainly cared for by their mothers (Table 3). Children whose mothers were aged 30–40 years (45 %), reported the highest frequencies in nearly all types of injuries except for swallowing sharp objects and sunstroke. Swallowing sharp objects and sunstroke were more common when the mother’s age was <30 years, accounting for 10.2 % (n = 56) and 2 % (n = 11) of cases, respectively. Almost three-quarters of the mothers had 10 or more years of full-time education (71.4 %) and the majority of injuries (70 %) were reported in this group. Children of working mothers had an incidence of all types of injuries 1.8-times higher than those of non-working mothers. These children were more likely to have wound (30.4 % vs. 35.5 %; p < 0.001) and fracture injuries (32 % vs. 26.7 %; p = 0.03) than those whose mothers were not working (Table 3). The child’s birth order was also a risk factor for childhood injuries (p < 0.0001). More than half of the injured children (53%) were ranked in second or third birth order in their families. This was positively correlated with fracture injuries (p = 0.03) (Table 3).

**Table 3:** Socioeconomic characteristics of study population

	Total	Wounds (451) % (n)	Fractures (422) % (n)	Burns (299) % (n)	Foreign body (124) % (n)	Poisoning (115) % (n)
Region						
Urban	862	31 (268)	29 (251)	19.6 (169)	9.2 (79)	7.7 (66)
Rural	610	30 (183)	28 (171)	21.3 (130)	7.4 (45)	8 (49)
P value		0.688	0.682	0.431	0.253	0.844
Child order						
1st	491	29.8 (146)	29 (143)	18.8 (92)	9.6 (47)	7.3 (36)
2nd- 3rd	780	32.2 (251)	29.5 (230)	20 (156)	7.7 (60)	7.2 (56)
≥ 4th	201	31.3 (63)	20.4 (41)	25.4 (51)	6.5 (13)	7.5 (15)
P value		0.513	0.032	0.137	0.316	0.989
Maternal age (yr)						
< 30	547	32.2 (176)	26.3 (144)	21.8 (119)	10.2 (56)	8.4 (46)
30- < 40	631	32.9 (201)	32.3 (204)	21.2 (134)	7.9 (50)	8.7 (55)
≥ 40	221	33.5 (74)	33.5 (74)	20.8 (46)	8.2 (18)	6.3 (14)
P value		0.901	˂0.001	0.953	0.348	0.529
Maternal education						
Illiterate	135	27.4 (37)	30.4 (41)	28.9 (39)	5.2 (7)	9.6 (13)
< 2ry	265	32.8 (87)	38.9 (103)	12.8 (34)	14.3 (38)	9 (24)
≥ 2ry	999	32.7 (327)	27.8 (278)	22.6 (226)	7.9 (79)	7.8 (78)
P value		0.450	0.002	0.002	0.001	0.662
Maternal working for cash						
Working	898	30.4 (273)	32 (288)	21.8 (196)	8.2 (74)	8 (73)
Not working	501	35.5 (323)	26.7 (134)	20.6 (103)	10 (50)	8.4 (42)
P value		˂0.001	0.039	0.587	0.281	0.919

### Knowledge, attitude and practice (KAP) among primary carers

**Fig. 2:**
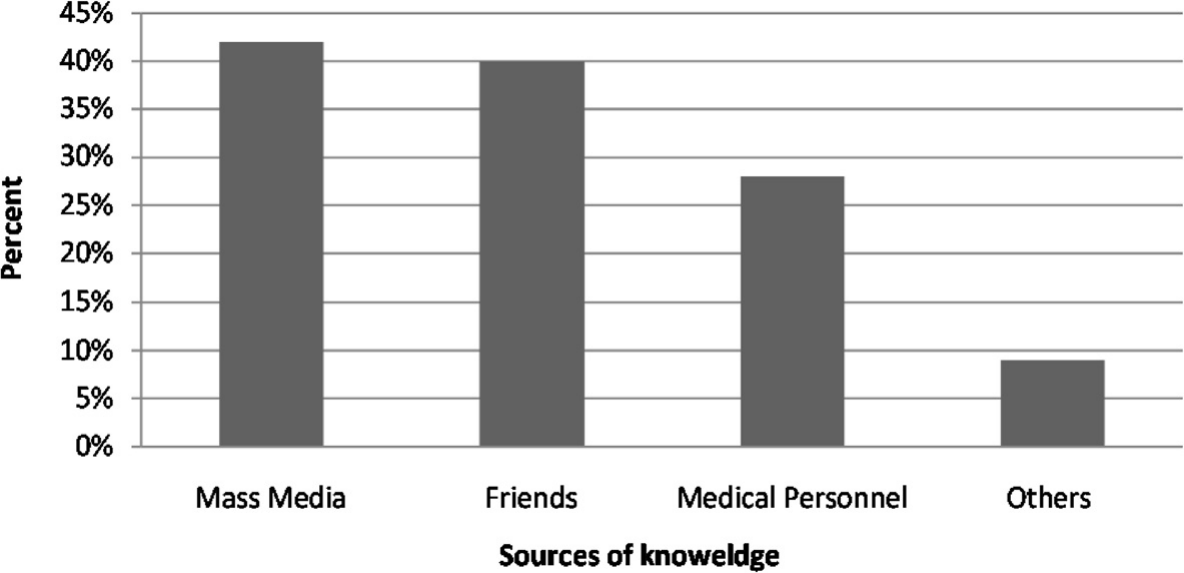
Sources of knowledge about childhood injury management

Only 37.7 % (n = 527) of the carers were familiar with the term “first aid”. The main source of knowledge about first aid was mass media (television and/or radio), reported by 42 % (n = 221) of carers (Fig. 2).

The attitude of the child’s carer towards the “importance of first aid” was also investigated. Carers (n = 1049, 75 %) agreed that mothers or primary carers should know about first aid for childhood injuries. Only 42.6 % (n = 596) of the carers felt that training was required to offer appropriate first aid, and only 12.5 % (n = 175) of participants were willing to undergo such a training course. Carers were classified into groups according to their responses for the management of each type of injury (Table 4).

**Table 4:** Practice of the primary carers about management of non-intentional childhood injuries

	Go to hospital % (n)	Traditional procedure-based therapies % (n)	Proper first aids % (n)	Don't know % (n)
Wounds	15.3 (214)	41 (573)	13.2 (134)	30.5 (428)
Fractures	22.1 (309)	91.1 (276)	4.3 (60)	53.9 (754)
Burn	18.6 (206)	21.7 (304)	6.6 (92)	53.1 (743)
Drowning	52 (727)	9 (127)	2.7 (37)	36.3 (508)
Poisoning	37 (518)	16.7 (233)	4.7 (66)	41.6 (582)
Suffocation	15.5 (217)	10.4 (146)	6.2 (87)	67.9 (949)
P value	< 0.001	< 0.001	< 0.001	< 0.001

## Discussion

Our study revealed specific patterns of unintentional childhood injuries based on age, gender, and location. When age-specific injury rates were studied, children aged 2–6 years had the highest overall injury rate (70 %). At virtually all ages, boys had higher tendency for all types of injuries than girls except for the swallowing of foreign bodies. These findings are consistent with other studies conducted in Egypt [23] as well as elsewhere in the world [24–27].

Overall, our results are consistent with previously published research on accidental childhood injuries [28, 29], with predominance of injuries related to falls, burns and RTIs. Similar to other studies [30, 31], falls were the leading cause of injury among all age groups, frequently resulted in fractures and wounds. Lack of supervision, inadequate safety standards for household furniture and goods, limited access to safe play areas, and uneven walking surfaces have been reported as risk factors for childhood falls in developing countries [29]. This suggests that more studies are needed for better understanding of the context in which trauma and injury occur in LMICs [32].

Age and gender-stratified analysis has raised concerns regarding prevention programmes targeting specific groups of children. For example, children under 2 years of age and in the home setting were associated with higher probability of burn injuries resulting from hot water, liquids, and flame. Similar results were reported from Ghana [33], Bangladesh [34], and Pakistan [35]. This pattern is probably similar in most undeveloped countries in which people rely heavily on floor-level and open fire cooking [36]. Moreover, young children and adolescents are at higher risk of sustaining RTIs than other age groups, with the peak injury ages being 3.5 and 12 years. Similar results were observed in a study carried out in South Africa, where the peak age for RTIs was 5–6 years [37]. These findings emphasise the necessity of implementing the Safe Systems Approach in child road safety in Egypt [38].

Consistent with previous work from the Arabian Gulf [39], we reported more frequent poisoning among children aged 2–6 years than other age groups, predominantly with kerosene and medications. In developing countries, unsafe storage of poisons in households; puts infants and young children at high risk for accidental ingestion [34]. We also found that poisoning was more common in urban settings. This differed from the results seen in LMICs [34]. This might be attributable to sampling bias because a larger proportion of our sample was located in urban communities compared with previous surveys.

In the present study, a few children who drowned or nearly drowned were almost identified in rural settings. Although our results were consistent with those of another Egyptian study [28], however, several earlier studies have shown higher rates of drowning [40, 41]. This difference may be partially explained by the fact that most drowning victims in LMICs die before reaching health facilities [6]. The small number of carers who reported supervising the children while they were near water suggests that one area of intervention should include education programmes for parents, which focus on close and constant supervision [6].

Our research highlights the importance of parents and carers making their homes safe for young children [28]; since more than 60 % of injuries occurred in the home setting. Studies from Trinidad and Tobago, Ethiopia and Nigeria [42–44] found similar results. In contrast to several previous researches [8, 45], we reported a substantial number of children sustain a higher incidence of injuries in urban than in rural settings. However, studies in Canada [46] and Europe [47] found findings similar to those in our study. Factors that might have influenced the differences may be related to limited access to medical facilities in rural areas, especially in Upper Egypt, and the relatively higher number of urban participants in our study sample. Health insurance coverage may be another explanation; as a larger proportion of rural residents are uninsured and thus may not have sought treatment [48].

Our study provides insight into maternal socioeconomic factors as determinants of childhood injuries in Egypt. Maternal age, education, working status and a child’s birth order had strong effects on childhood injuries. We found that children of mothers with a high educational level had a more than twofold increased risk for almost all types of injuries. Other previous studies showed different results [26, 49]. This difference may be attributable to the high number of children with highly educated mothers and a weaker health infrastructure, which may facilitate the ability of more educated women to seek medical consultation. We also found that maternal age (≥30 years) was significantly associated with an increased incidence of fracture injuries, a result that differed from a Bangladeshi study that showed the children of older mothers were less likely to have injury morbidity than the children of younger (<25 years) mothers [50]. This might be explained by differences in cultures and sample size. In our study, children with working mothers had higher incidence of childhood injuries than those with non-working mothers, except for wounds. These findings are an important reminder that child health may be affected by maternal time allocation and by the quality of non-maternal child care [51]. Likewise, second and third birth order was associated with increased incidence of fracture injuries. This is important; as children lacking proper supervision by their parents with more supervision by older siblings have a higher risk of childhood injuries [1, 52].

The significant findings in this community-based injury survey in Egypt add to the existing literature on childhood injuries. This is the first study that utilized a child population with a wide age range (0–18 years) as well as a varied type of injury presenting a view of the magnitude and frequency of accidental injuries seen in urban and rural Egyptian communities. In many developing countries, injury cases are often treated outside hospitals, and many health facilities lack proper record keeping systems. Therefore, household surveys can provide more representative samples of population-based injury data compared with hospital records [26]. Nonetheless, important study limitations should be noted. Our data were based on household interviews and thus subject to recall bias. Moreover, this method used in the data collection may have overestimated the number and incidence of injuries, because participants might have reported older injuries as well as those occurring during the last 6 months. This is a “telescoping effect”, that is, a tendency to remember events as occurring closer to the present than they actually occurred [35]. Decreasing the ultimate sample size from one thousand nine hundred seventy seven to one thousand three hundred ninety nine limited the power of generalisability of our results. Therefore, assumption about population-based injury patterns experienced by young people should be made cautiously. Furthermore, we were restricted in our ability to assess the age-related risk factors for all unintentional injuries of the age group 12–18 years; as this group represented very small percentage of the total study sample. Finally, we were not able to assess all of the socioeconomic risk factors known to be associated with an increased risk of injuries such as family income, fathers’ education level and occupation because the culture and traditions of the Egyptians, particularly in rural communities, mean they are conservative in providing such information.

### Implications

This study identified the types, circumstances and possible risk factors for non-intentional childhood injuries in Egypt. Our findings suggest that prevention activities by the Egyptian government should prioritise reducing childhood injuries caused by falls, burns and RTIs. Our data may also improve understanding of how different types of injuries correlate with sex, age and socioeconomic status. Future research should consider whether certain programmes or interventions are more or less effective for certain subgroups of children. We also examined the general attitudes and beliefs of parents towards childhood injuries. These data may be useful in implementing effective, family-level interventions. Prevention measures should consider strategies targeted to different injury settings: the home, school and the street. Our study highlighted the need for large-scale, longitudinal surveys to comprehensively investigate accidental injuries and obtain data to assess aetiology, correlations and consequences of different injury outcomes.

## Conclusions

Childhood injuries represent a substantial public health problem in LMICs. Boys had a higher injury incidence rate than girls, and falls were the most common type of injury among all age groups. Home and its surroundings was the most common setting for injuries, and children were usually injured during indoor play. Maternal socioeconomic characteristics were also strongly associated with childhood injuries. Therefore, we need to promote actions to reduce the rate of injuries among our children by considering these results. Such actions should be based on scientific evidence and also consider the practical aspects related to implementing injury prevention programmes in Egypt.
